# Spontaneous sputum conversion and reversion in *Mycobacterium abscessus* complex lung disease

**DOI:** 10.1371/journal.pone.0232161

**Published:** 2020-04-27

**Authors:** Kyung-Wook Jo, Yea Eun Park, Yong Pil Chong, Tae Sun Shim

**Affiliations:** 1 Division of Pulmonology and Critical Care Medicine, Department of Internal Medicine, University of Ulsan College of Medicine, Asan Medical Center, Seoul, South Korea; 2 Department of Infectious Diseases, University of Ulsan College of Medicine, Asan Medical Center, Seoul, South Korea; National Institute of Infectious Diseases, JAPAN

## Abstract

**Background:**

We aim to investigate the rate of spontaneous sputum conversion and reversion in patients with *Mycobacterium abscessus* complex (MABC) lung disease.

**Methods:**

Among 241 patients diagnosed with MABC lung disease between July 2012 and December 2018, 126 patients with persistent sputum positivity for ≥ 6 months without treatment were enrolled at a tertiary referral center in South Korea. Patients were subdivided into two groups, depending on whether or not treatment was initiated within 2 years of diagnosis. The rates of spontaneous sputum culture conversion and reversion was investigated in patients who did not receive treatment within 2 years.

**Results:**

The mean age of 126 patients was 62.9 years. During a mean follow-up duration of 3.2 years, 33 (26.2%) patients received treatment within 2 years of diagnosis. Among the remaining 93 patients not receiving treatment within 2 years, spontaneous sputum conversion occurred in 24 (25.8%) patients during a mean follow-up duration of 3.7 years after diagnosis. No significant differences were observed in time to conversion between *Mycobacterium abscessus* and *Mycobacterium massiliense* lung diseases. The Cox regression analysis showed that malignancy as a comorbid disease and the lower number of lobes involved were independent predictors of spontaneous sputum conversion. After spontaneous sputum conversion, reversion occurred in 27.8% patients at a median of 18.2 months after conversion.

**Conclusions:**

Among patients with MABC lung disease who did not receive treatment for at least 2 years after diagnosis, approximately one-fourth experienced spontaneous conversion. However, not a few patients experienced reversion after spontaneous conversion.

## Introduction

*Mycobacterium abscessus* complex (MABC) is a rapidly growing mycobacterium associated with several diseases in humans, of which lung disease is the most common [1]. It is the second most common cause of nontuberculous mycobacteria (NTM) pulmonary disease in many countries including South Korea, after *Mycobacterium avium* complex (MAC) [2,3]. MABC comprises three different subspecies, namely, *M*. *abscessus* subsp. *abscessus* (hereafter referred to as *M*. *abscessus*) and *M*. *abscessus* subsp. *massiliense* (hereafter referred to as *M*. *massiliense*), and *M*. *abscessus* subsp. *bolletii* (hereafter referred to as *M*. *bolletii*), which has been determined on the basis of gene sequence analysis [4]. In South Korea, *M*. *bolletii* represents only 1% of the clinical strains of MABC, whereas *M*. *abscessus* and *M*. *massiliense* account for approximately half of the causative organims of MABC [5,6]. Although the virulence of *M*. *abscessus* and *M*. *massiliense* may not differ in terms of disease progression rate [6], treatment outcomes are much better in patients with *M*. *massiliense* lung disease than in those with *M*. *abscessus* lung disease [5,7].

One peculiar feature of MAC lung disease is that a substantial portion of untreated patients with stable course can experience spontaneus sputum conversion. Previously, we showed that spontaneous conversion of sputum cultures occurred in approximately half the 93 untreated patients with MAC lung disease [8]. Furthermore, we observed that spontaneous sputum conversion occurred in 106 (52.2%) of 203 patients with noncavitary nodular bronchiectatic (NC-NB) type disease who had not received treatment, during a median follow-up duration of 5.0 years [9]. Another study in South Korea found that 34% of patients with NTM lung disease who did not receive treatment showed spontaneous culture conversion [10]. This tendency of spontaneous conversion is one of the main reasons for not recommending immediate treatment initiation for many patients with indolent and mild NC-NB-type MAC lung disease.

Although MABC is the second most common causative agent of NTM lung disease in South Korea, there has been no research regarding whether spontaneous culture conversion specifically occurs in patients with MABC lung disease. In addition, if spontaneous culture conversion does occur in patients with MABC lung disease, its predictors and clinical course after spontaneous culture conversion remain to be elucidated. We therefore aimed to investigate these issues in this study.

## Materials and methods

## Study subjects

Patients were enrolled from Asan Medical Center, which is a 2,700-bed referral hospital in Seoul, South Korea. Between July 2012 and December 2018, 241 patients were identified who met the American Thoracic Society criteria for MABC lung disease. After excluding patients (i) who received treatment within 6 months of diagnosis, (ii) whose follow-up duration after diagnosis was <6 months, (iii) with insufficient data, including those for sputum examinations, (iv) who had received surgical resection, and (v) with spontaneous conversion occurring within 6 months of diagnosis, the medical records of the remaining patients with persistent sputum positivity for ≥6 months were retrospectively analyzed in July 2019. Only patients in whom spontaneous sputum examinations were performed at least once every 6 months were included in the analysis.

The study protocol was approved by the institutional review board of the Asan Medical Center (IRB No.: 2019–0938). The board waived the requirement for informed consent because of the retrospective nature of the analysis.

### Patient group

Patients with persistent sputum positivity for ≥6 months without treatment were first classified into two groups based on whether or not the patients received treatment within 2 years of diagnosis. A cut-off period of 2 years was chosen because a previous study in South Korea reported that patients with MABC lung disease initiated treatment, on an average, approximately 2 years after diagnosis [6].

The sputum culture conversion rate was investigated for patients who did not receive treatment within 2 years of diagnosis. We defined spontaneous sputum conversion as three consecutive negative cultures spanning at least a 2-month period, with the time of conversion defined as the date of the first negative culture [11]. Among patients who experienced spontaneous sputum conversion, we analyzed if reversion occurred in those who were followed up for ≥6 months after conversion. Reversion was defined as two consecutive positive MABC cultures after sputum conversion [11].

### Radiologic evaluation

Radiographic anomalies upon chest computed tomography at the time of diagnosis were classified into NC-NB and other radiologic types (the sum of fibrocavitary, cavitary nodular bronchiectatic, and unclassifiable type), of which each radiologic type was previously defined [12,13]. The extent of infiltration was determined according to the number of lobes involved. Lingula division was regarded as a separate lobe, leading to a total of six lung lobes that were considered for analysis [9].

### Microbiological examination

Expectorated sputum or bronchoscopy samples were cultured in both solid (Ogawa medium; Korean Institute of Tuberculosis, South Korea) and liquid (BACTEC 960 Mycobacterial Growth Indicator Tube; Becton Dickinson, Sparks, MD, USA) mediums. Acid-fast bacillus (AFB) smears were prepared via Ziehl–Neelsen staining. Positive liquid cultures and colonies on solid medium were subjected to polymerase chain reaction (PCR) using the Seeplex TB detection system (Seegen, Seoul, Korea) to differentiate between *Mycobacterium tuberculosis* complex and NTM. The species of NTM isolates were identified via PCR and restriction fragment length polymorphism methods based on the *rpoB* gene or via reverse blot hybridization assays of *rpoB* [14]. Differentiation between *M*. *abscessus* and *M*. *massiliense* was performed based on analysis of the *erm*(41) gene detected by PCR [15].

Drug susceptibility test (DST) was performed using the broth microdilution method [16]. Due to the poor solubility of azithromycin at its high concentrations, determining the minimal inhibitory concentration (MIC) of azithromycin is considered to be difficult, so instead the MIC of clarithromycin was used to represent the MIC of azithromycin. The MIC of clarithromycin was determined on days 3 and 14 after incubation; MABC isolates were considered susceptible (MIC ≤ 2 μg/mL at days 3 and 14), resistant (MIC ≥ 8 μg/mL at day 3), or inducibly resistant (susceptible at day 3 but resistant at day 14) to clarithromycin [16].

### Statistical analyses

All data are presented as means (± standard deviations) or medians [interquartile range (IQR)] for continuous variables, and numbers (%) for categorical variables. Data were compared via Student’s t-tests for continuous variables and χ2 or Fisher’s exact tests for categorical variables. All tests were two-sided and P values < 0.05 were considered statistically significant. The spontaneous conversion rates after diagnosis between the two species and radiologic types were compared using Kaplan–Meier method. The Cox regression analysis was used to calculate the adjusted hazard ratio (HR) and 95% confidence intervals (CIs) to predict spontaneous conversions. The variables used in the Cox analysis were those that had a statistical significance level of <0.05 in the univariate analysis. All data were analyzed using SPSS software (version 24.0; SPSS, Chicago, IL).

## Results

### Study subjects

**Fig 1:**
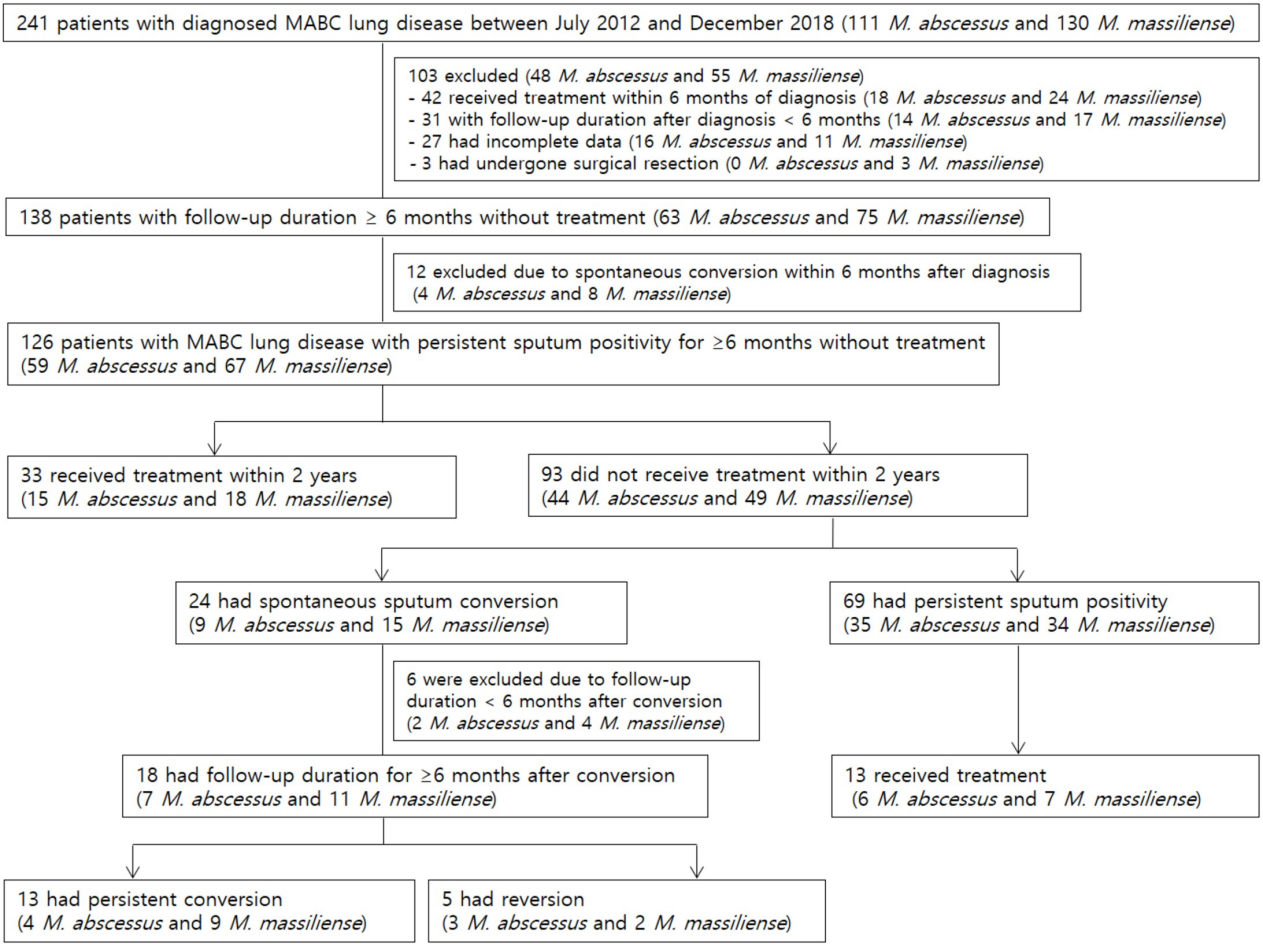
Study flow chart. MABC, *Mycobacterium abscessus* complex.

Eligibility screening identified 126 patients with MABC lung disease with persistent sputum positivity for ≥6 months (Fig 1), of which 59 had *M*. *abscessus* lung diseases and the remaining 67 had *M*. *massiliense* lung diseases. The mean age of the patients was 62.9 years, and 64.3% were female. NC-NB type was the most common radiological type that was noted in 67.5% (85/126) of patients. During a mean follow-up duration of 3.2 ± 1.7 years, 33 (26.2%) patients received treatment within 2 years of diagnosis, whereas the remaining 93 (73.8%) did not. The mean follow-up durations after diagnosis were indifferent for patients who did and who did not receive treatment within 2 years (3.3 ± 1.6 and 3.1 ± 1.7 years, respectively; *P* = 0.579) Except for the higher rates of lung disease with cavity and radiologic type other than NC-NB in the patients who received treatment within 2 years, the clinical characteristics of the two groups were generally comparable (Table 1).

**Table 1:** Clinical characteristics of 126 patients with *Mycobacterium abscessus* complex lung disease based on whether treatment was initiated within 2 years of diagnosis.

Characteristics	Total (n = 126)	Treatment within 2 years (n = 33)	No treatment within 2 years (n = 93)	*P* value
Age (years)	62.9 ± 10.9	63.6 ± 11.7	62.6 ± 10.6	0.677
Female gender	81 (64.3%)	19 (57.6%)	62 (66.7%)	0.349
Body mass index (kg/m^2^)	20.8 ± 2.6	20.4 ± 2.3	21.0 ± 2.7	0.247
Current or past smoker	30 (23.8%)	10 (30.3%)	20 (21.5%)	0.308
Diagnosis made by bronchoscopy	9 (7.1%)	2 (6.1%)	7 (7.5%)	> 0.99
Previous history of TB treatment	44 (34.9%)	12 (36.4%)	32 (34.4%)	0.840
Previous history of NTM treatment				0.465
*Mycobacterium avium* complex	31 (24.6%)	8 (24.2%)	23 (24.7%)	
*Mycobacterium kansasii*	3 (2.4%)	1 (3.0%)	2 (2.2%)	
*Mycobacterium abscessus* complex	2 (1.6%)	1 (3.0%)	1 (1.1%)	
*Mycobacterium fortuitum*	1 (0.8%)	1 (3.0%)	0	
Comorbidities				
Bronchiectasis	38 (30.2%)	6 (18.2%)	32 (34.4%)	0.081
Malignancy	13 (10.3%)	3 (9.1%)	10 (10.8%)	> 0.99
Chronic obstructive lung disease	12 (9.5%)	3 (9.1%)	9 (9.7%)	> 0.99
Chronic liver disease	11 (8.7%)	4 (12.1%)	7 (7.5%)	0.477
Diabetes mellitus	10 (7.9%)	2 (6.1%)	8 (8.6%)	> 0.99
Interstitial lung disease	7 (5.6%)	3 (9.1%)	4 (4.3%)	0.379
Chronic kidney disease	2 (1.6%)	0	2 (2.2%)	> 0.99
Use of immunosuppressant	2 (1.6%)	0	2 (2.2%)	> 0.99
Etiology				0.854
*Mycobacterium abscessus*	59 (46.8%)	15 (45.5%)	44 (47.3%)	
*Mycobacterium massiliense*	67 (53.2%)	18 (54.5%)	49 (52.7%)	
Isolation of other microbiological organisms				
Other NTM species	29 (23.0%)	9 (27.3%)*	20 (21.5%)^†^	0.499
Bacterial or Fungal organisms	20 (15.9%)	5 (15.2%)^‡^	15 (16.1%)^§^	0.865
Radiologic type				0.023
Noncavitary nodular bronchiectatic	85 (67.5%)	17 (51.5%)	68 (73.1%)	
Other radiologic types^¶^	41 (32.5%)	16 (48.5%)	25 (26.9%)	
Pulmonary function test (n = 93)				
Prebronchodilator FEV_1_ (% pred)	73.7 ± 23.2	71.2 ± 27.4	74.6 ± 21.4	0.525
Prebronchodilator FVC (% pred)	77.8 ± 17.3	73.4 ± 16.1	79.4 ± 17.6	0.131
Positive AFB smear at treatment initiation	76 (60.3%)	24 (72.7%)	52 (55.9%)	0.090
The presence of cavity	32 (25.4%)	13 (39.4%)	19 (20.4%)	0.032
Number of involved lobes	3.4 ± 1.5	3.7 ± 1.3	3.2 ± 1.6	0.071

Data are reported as mean ± standard deviations or number (%).

TB, tuberculosis; NTM, nontuberculous mycobacterium; AFB, acid-fast bacilli; FEV_1_: forced expiratory volume in 1 second; % pred: percentage of predicted value; FVC: forced vital capacity.

*Isolated species in detail were as follows: *Mycobacterium avium* (n = 7), *Mycobacterium intracellulare* (n = 2)

^†^Isolated species in detail were as follows: *Mycobacterium avium* (n = 11), *Mycobacterium intracellulare* (n = 6), *Mycobacterium chelonae* (n = 2), *Mycobacterium fortuitum* (n = 1)

^‡^Isolated microbiologic organisms in detail were as follows: *Pseudomonas aeruginosa* (n = 3), *Klebsiella pneumoniae* (n = 1), *Stenotrophomonas maltophilia* (n = 1)

^§^Isolated microbiologic organisms in detail were as follows: *Pseudomonas aeruginosa* (n = 7), *Klebsiella pneumoniae* (n = 3), *Aspergillus fumigatus* (n = 3), *Staphylococcus aureus* (n = 1), *Acinetobacter baumanni* (n = 1)

^¶^Cavitary nodular bronchiectatic (n = 18), fibrocavitary (n = 14) and unclassifiable type(n = 9)

DST was performed for 99 patients (51 *M*. *abscessus* and 48 *M*. *massiliense*). Among the 99 patients, one *M*. *massiliense* isolate was resistant to clarithromycin. Of the remaining 98 isolates susceptible to clarithromycin, inducible resistance was observed in 84.3% (43/51) of isolated *M*. *abscessus* strains and 6.4% (3/47) of *M*. *massiliense* strains.

### Spontaneous sputum conversion and predictor

**Fig 2:**
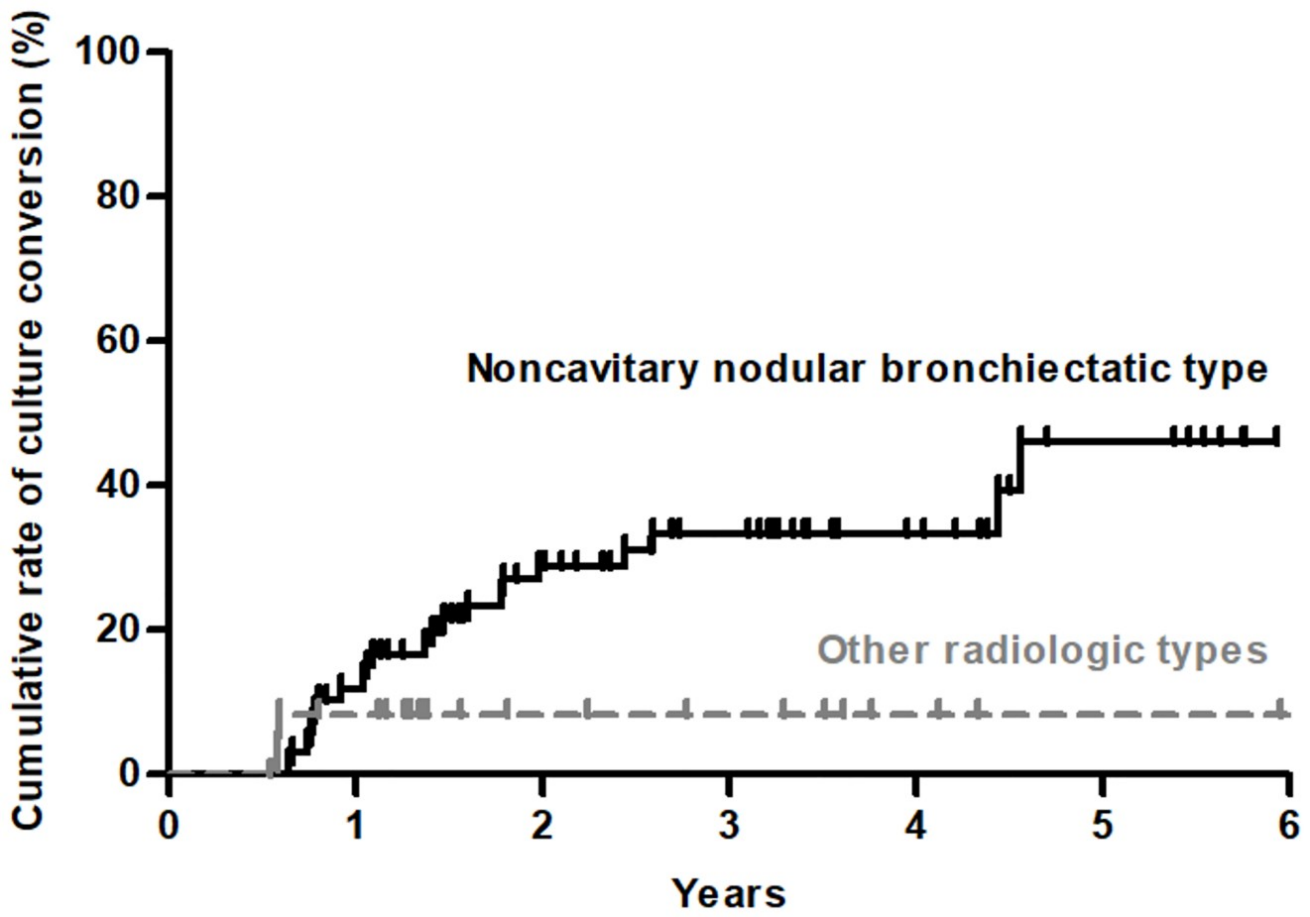
Kaplan–Meier estimates of the cumulative rates of sputum culture conversion between noncavitary nodular bronchiectatic type and other radiologic types (fibrocavitary, cavitary nodular bronchiectatic, and unclassifiable types) (*P* = 0.187 by Gehan–Breslow test).

**Fig 3:**
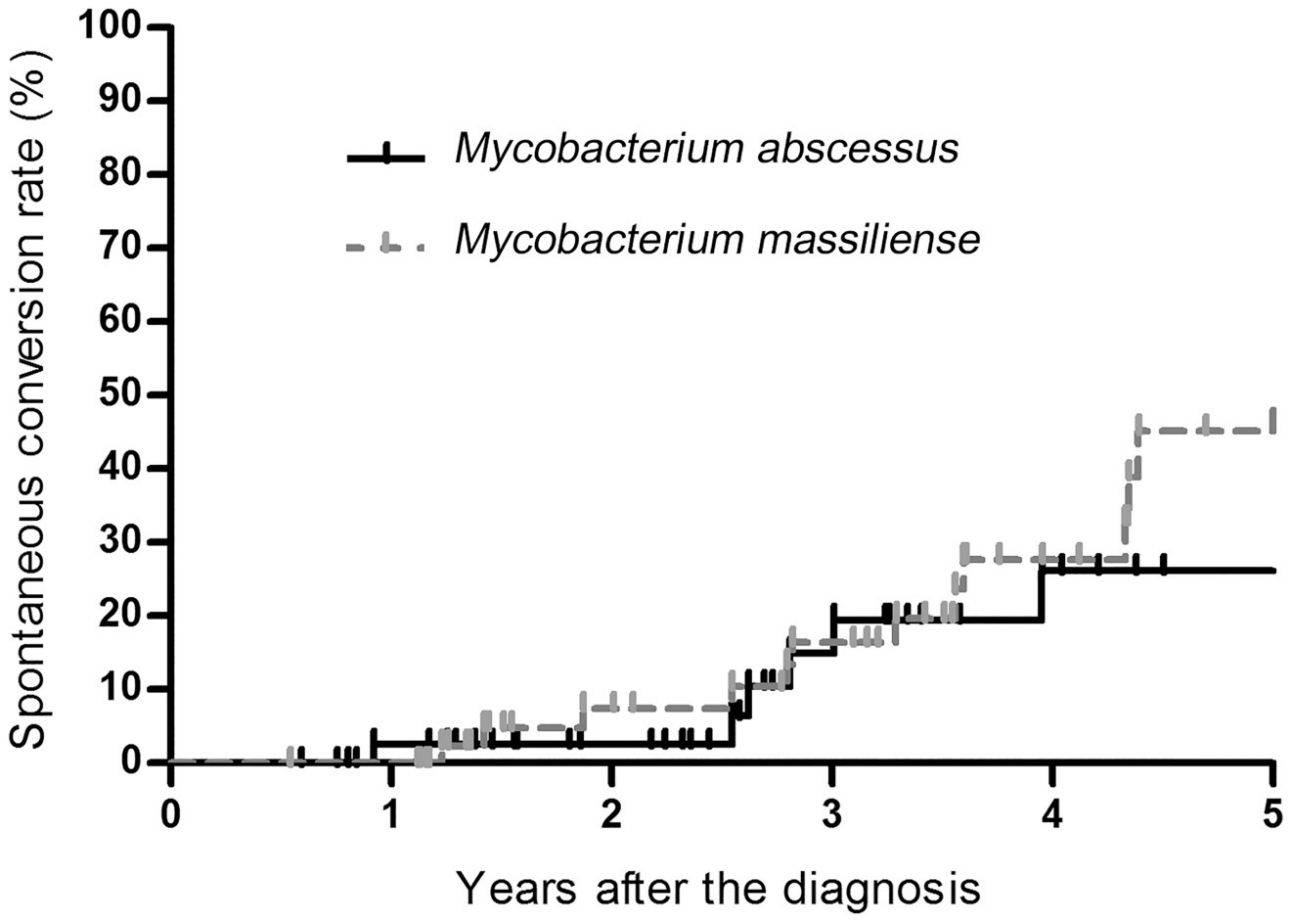
Kaplan–Meier analysis of the comparison of time to spontaneous sputum culture conversion between *Mycobacterium abscessus* and *Mycobacterium massiliense* lung diseases (*P* = 0.590 by Gehan–Breslow test).

Analysis of 93 patients who received no treatment within 2 years revealed that spontaneous sputum conversion occurred in 24 (25.8%) of these patients during a mean follow-up duration of 3.7 ± 1.7 years, whereas the remaining 69 (74.2%) patients had persistent sputum culture positivity during a mean follow-up duration of 2.9 ± 1.6 years. For all 93 patients, sputum specimens were collected, on an average, 8.3 ± 5.7 times per patient during the follow-up period for AFB culture. Specimens were collected 6.5 ± 6.1 times for the 24 patients with spontaneous conversion and 8.9 ± 5.5 times for the 69 patients with persistent sputum positivity (*P* = 0.075). Fig 2 shows the Kaplan–Meier estimates of the cumulative rates of sputum culture conversion according to the radiologic type; the sputum culture conversion rates in the two groups were similar. In addition, no significant differences were observed in the time to spontaneous sputum culture conversion between *M*. *abscessus* and *M*. *massiliense* lung diseases by Kaplan–Meier analysis (*P* = 0.590, Fig 3).

As shown in Table 2, there were significant differences in terms of malignancy as a comorbid disease, radiologic type, and the number of lobes involved in MABC lung disease between the two groups according to the spontaneous conversion. Cox regression analysis showed that malignancy (adjusted HR, 2.664; 95% CI, 1.038–6.834; *P* = 0.042) and the number of involved lobes (adjusted HR, 0.677; 95% CI, 0.489–0.937; *P* = 0.019) were independent predictors of spontaneous sputum conversion among these patients (Table 3).

**Table 2:** Clinical characteristics of 93 patients with *Mycobacterium abscessus* complex lung disease who did not receive treatment within 2 years of diagnosis based on spontaneous sputum conversion.

Characteristics	Spontaneous sputum conversion (n = 24)	Persistent sputum positivity (n = 69)	*P* value
Age (years)	61.4 ± 11.1	63.0 ± 10.5	0.522
Female gender	15 (62.5%)	47 (68.1%)	0.615
Body mass index (kg/m^2^)	21.1 ± 2.7	21.4 ± 5.1	0.781
Current or past smoker	7 (29.2%)	13 (18.8%)	0.289
Diagnosis made by bronchoscopy	3 (12.5%)	4 (5.8%)	0.369
Previous history of TB treatment	8 (33.3%)	24 (34.8%)	0.898
Previous history of NTM treatment			0.689
*Mycobacterium avium* complex	5 (20.8%)	18 (26.1%)	
*Mycobacterium kansasii*	0	2 (2.9%)	
*Mycobacterium abscessus* complex	0	1 (1.4%)	
Comorbidities			
Bronchiectasis	7 (29.2%)	25 (36.2%)	0.530
Malignancy	6 (25.0%)	4 (5.8%)	0.017
Chronic obstructive lung disease	2 (8.3%)	7 (10.1%)	> 0.99
Diabetes mellitus	2 (8.3%)	6 (8.7%)	> 0.99
Chronic liver disease	4 (16.7%)	3 (4.3%)	0.070
Interstitial lung disease	1 (4.2%)	3 (4.3%)	> 0.99
Chronic kidney disease	1 (4.2%)	1 (1.4%)	0.452
Use of immunosuppressant	2 (8.3%)	0	0.065
Etiology			0.264
*Mycobacterium abscessus*	9 (37.5%)	35 (50.7%)	
*Mycobacterium massiliense*	15 (62.5%)	34 (49.3%)	
Isolation of other microbiological organisms			
Other NTM species	6 (25.0%)*	14 (20.3%)^†^	0.629
Bacterial or Fungal organisms	4 (16.7%)^‡^	11 (15.9%)^§^	> 0.99
Radiologic type			0.017
Noncavitary nodular bronchiectatic	22 (91.7%)	46 (66.7%)	
Other radiologic types^¶^	2 (8.3%)	23 (33.3%)	
Pulmonary function test (n = 67)			
Prebronchodilator FEV_1_ (% pred)	79.4 ± 22.4	73.0 ± 21.1	0.291
Prebronchodilator FVC (% pred)	83.2 ± 21.9	78.2 ± 15.9	0.309
Positive AFB smear at treatment initiation	10 (41.7%)	42 (60.9%)	0.103
The presence of cavity	2 (8.3%)	17 (24.6%)	0.140
Number of involved lobes	2.4 ± 1.5	3.6 ± 1.5	0.001

Data are reported as mean ± standard deviations or number (%).

TB, tuberculosis; NTM, nontuberculous mycobacterium; AFB, acid-fast bacilli; FEV_1_: forced expiratory volume in 1 second; % pred: percentage of predicted value; FVC: forced vital capacity.

*Isolated species in detail were as follows: *Mycobacterium intracellulare* (n = 3), *Mycobacterium avium* (n = 2), *Mycobacterium chelonae* (n = 1)

^†^Isolated species in detail were as follows: *Mycobacterium avium* (n = 9), *Mycobacterium intracellulare* (n = 3), *Mycobacterium chelonae* (n = 1), *Mycobacterium fortuitum* (n = 1)

^‡^Isolated microbiologic organisms in detail were as follows: *Pseudomonas aeruginosa* (n = 2), *Klebsiella pneumoniae* (n = 1), *Staphylococcus aureus* (n = 1)

^§^Isolated microbiologic organisms in detail were as follows: *Pseudomonas aeruginosa* (n = 5), *Aspergillus fumigatus* (n = 3), *Klebsiella pneumoniae* (n = 2), Acinetobacter baumanni (n = 1)

^¶^Cavitary nodular bronchiectatic (n = 11), fibrocavitary (n = 8) and unclassifiable type (n = 6)

**Table 3:** Analyses of factors affecting spontaneous sputum conversion in 93 patients with noncavitary nodular bronchiectatic type *Mycobacterium abscessus* complex lung disease.

Predictor	Univariate analysis		Multivariate analysis	
	HR (95% CI)	*P* value	aHR (95% CI)	*P* value
Noncavitary nodular bronchiectatic type	3.262 (0.766–13.890)	0.110	1.503 (0.337–6.708)	0.593
Malignancy	3.074 (1.219–7.756)	0.017	2.664 (1.038–6.834)	0.042
Number of involved lobes	0.658 (0.480–0.902)	0.009	0.677 (0.489–0.937)	0.019

Abbreviations: HR, hazard ratio; CI, confidence interval; aHR, adjusted HR

### Reversion after spontaneous conversion

**Fig 4:**
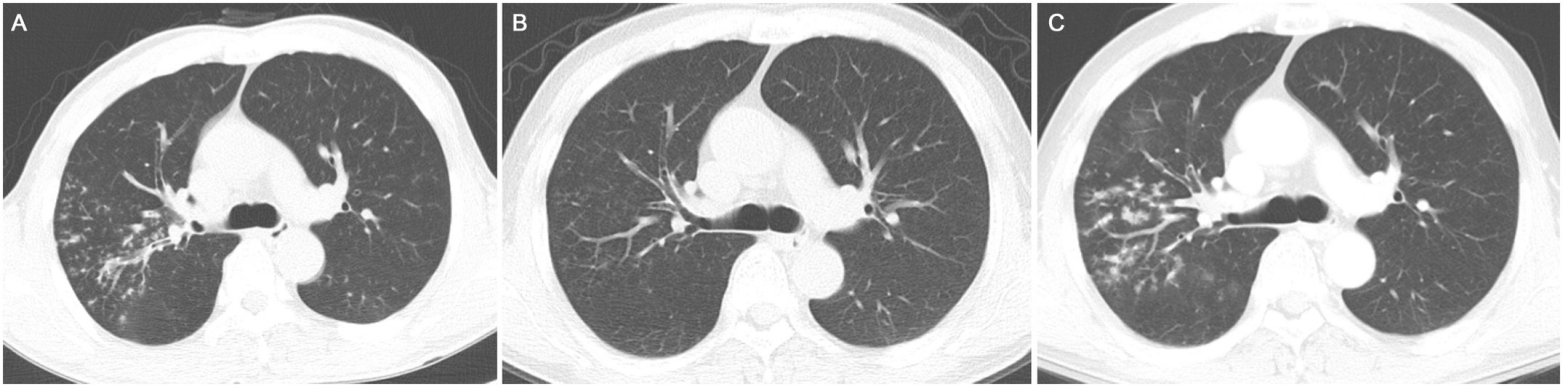
The changes in radiological imaging of one patient who experienced spontaneous conversion and reversion. (A) Chest computed tomography at the time of diagnosis of a 55 year-old man with *Mycobacterium abscessus* complex lung disease showing irregular centrilobular nodules with segmental distribution in the right upper lobe. (B) Follow-up chest computed tomography conducted approximately 16 months after diagnosis showing markedly improved radiological changes. The patient did not receive any treatment during the follow-up period and his sputum examination revealed spontaneous conversion. (C) Approximately 2 years later after spontaneous conversion, chest computed tomography showing redevelopment of centrilobular nodules in the right upper lobe. The growth of *Mycobacterium abscessus* complex was noted in the follow-up sputum examination.

Among the 24 patients who had spontaneous conversion, the follow-up duration after conversion was at least 6 months in 18 patients. Among these patients, reversion occurred in 5 (27.8%) patients at a median of 18.2 months (IQR, 13.2–35.4) after spontaneous sputum conversion (Fig 1). After spontaneous conversion, sputum AFB examination was performed 3.8 ± 1.6 and 4.2 ± 2.7 times during mean follow-up durations of 2.7 ± 1.5 and 2.9 ± 1.7 years for 13 patients with persistent conversion and five patients with reversion, respectively. All 18 patients spontaneously produced sputum without induction. No significant differences were observed in terms of baseline characteristics between the two groups (S1 Table). Fig 4 shows the changes in radiological imaging of one patient who experienced spontaneous conversion and reversion.

## Discussion

In contrast to MAC lung disease, in which spontaneous sputum conversion is known to occur in a considerable portion of patients [9,17], there is limited information of the clinical course in MABC lung disease patients who did not receive treatment. Although Moon et al. previously reported that spontaneous culture conversion occurred in 157 of 459 (34%) patients with NTM lung disease, the proportion of patients with MABC lung disease that had spontaneous culture conversion is unknown because they reported the conversion rate of patients with NTM lung disease for both MAC and MABC lung diseases together [10]. The most important finding of the present study was that, among patients with MABC lung disease with persistent sputum positivity for ≥6 months who did not receive treatment for at least 2 years after diagnosis, approximately 25% experienced spontaneous sputum conversion. The development of reversion in at least one-fourth of patients with spontaneous conversion is another important finding that we have found.

The treatment of MABC lung disease is challenging because of the necessity for prolonged intravenous therapy and frequent side effects [18,19]. In spite of this difficulty, the culture conversion rate for MABC lung diseases has been poor and reported to be 41.2% in a previous meta-analysis study, although it was signiticantly higher in *M*. *massiliense* than in *M*. *abscessus* [20]. The poorer treatment outcome of *M*. *abscessus* is related to a functional *erm*(41) gene, which confers inducible macrolide resistance [21]. In this regard, the findings of the present study imply that watchfully waiting for the possibility of spontaneous improvement could be a useful strategy for MABC lung disease management, particularly if the patient’s condition and the extent of disease does not require immediate treatment. Furthermore, this strategy can be considered if the extent of MABC lung disease inflitration is not substantial; the rate of spontaneous conversion was higher in cases with fewer lobes involved (Table 2).

Another notable finding of the present study was that there was no difference in the times from diagnosis to sputum conversion between *M*. *abscessus* and *M*. *massiliense* (Fig 3), which is consistent with a previous report that showed the rate of disease progression were indifferent between these two species during the follow-up period [6]. Additionally, there was no statistically significant difference in etiologic subspecies (*M*. *abscessus vs*. *M*. *massiliense*) between the patients with spontaneous sputum conversion and those with persistent positivity (Table 2). In addition, the proportion of MABC isolates with inducible resistance was similar between the two groups. All these findings suggested that the clinical course, such as spontaneous conversion or time to disease progression, was similar between the two subspecies.

Several factors could explain the phenomenon of spontaneous culture conversion in the patients with MABC lung disease. First, successful eradication by the induction of the host immune response could result in transient infection in patients with MABC lung disease [22], particularly in those with less severe lung involvement. In addition, considering one of the sources of MABC infection is the environment, such as from household plumbing systems [2,23,24], temporary exposure to such environmental sources could be another plausible explanation. Lastly, because local human-to-human transmission of MABC through fomite or long-lived infectious aerosols is possible [25], temporary exposure in a hospital environment may lead to a transient and spontaneously improved lung disease. However, it should be noted that not a few patients with MABC lung disease experienced reversion after spontaneous conversion in the present study. A previous report has also shown that 17% (26/157) of patients with spontaneous culture conversion redeveloped NTM lung disease caused by a different NTM species [10]. These findings imply that continuous and lifetime follow-up is necessary for patients with NTM lung disease, even those who experience spontaneous sputum conversion.

Of the 241 patients with MABC lung disease in this study, 32.4% (78/241), including 42 who received treatment within 6 months after diagnosis, 33 who received treatment within 2 years, and 3 who underwent surgical resection, received treatment for MABC lung disease within 2 years. This rate of 32.4% is lower than that shown in previous studies reported in other centers of South Korea. For example, Koh *et al*. reported that 93 of 167 patients (55.6%) with *M*. *abscessus* lung disease underwent antibiotic treatment [12]. Moreover, another study revealed that disease progression that required treatment was noted in 37.5% (21/56) of patients with *M*. *abscessus* lung diseases and 38.9% (21/54) of those with *M*. *massiliense* lung diseases [6]. Although the reason for this discrepancy is uncertain, a plausible explanation could be that the period in which patients were diagnosed with lung disease was different in each study. That is, the time of diagnosis for enrolled patients was between 2002 and 2012 in one study [12] and 2006 and 2015 in another [6]. Compared with these studies, we selected subjects whose diagnosis was made between July 2012 and December 2018. Considering studies showing unsatisfactory treatment outcomes (approximately 50% rate of culture conversion), even with the prolonged course of multiple antibiotic therapies, were published in 2009 [26] and 2011 [27], the attending physician during the study period may be reluctant to initiate treatment for patients with MABC lung disease.

Among our study subjects, malignancy and the lower number of lobes involved were independent predictors of spontaneous sputum conversion. The possible reason for the higher number of patients with spontaneous conversion who had malignancy as a comorbid disease was that the transient MABC infection that was associated with a weakened immunity owing to the malignancy itself or anti-cancer therapy [28] spontaneously improved as the immunity recovered after the cessation of the treatment of the malignancy. In addition, it was plausible that the mean number of lobes involved was significantly lower in patients with spontaneous conversion because spontaneous conversion is most likely to occur in patients who had a milder disease.

MABC manifests as two distinct phenotypic colony morphotypes: smooth and rough [29,30]. Considering MABC lung disease caused by the rough morphotype, as opposed to the smooth type, shows a more chronic and invasive phenotype [31,32], and is more refractory to antibiotic therapy [12], it is possible that the rates of spontaneous sputum culture conversion and reversion could be associated with the morphotype of MABC. However, we did not identify the macroscopical colony morphotype of MABC isolates in the majority of our patients because this information was not routinely reported in our center during the study period. We were able to identify the colony morphotype in only 3 of the 33 patients who received treatment within 2 years; 1 patient had the rough morphotype, and 2 had the smooth colony morphotype.

The present study had other several limitations of note. Most prominently, the analysis was conducted at a single referral center with a limited number of patients. Small number of patients included in the present study limited the statistical power of the analysis and the interpretation of the findings In addition, decisions regarding treatment initiation, follow-up interval, and requests for sputum examination were mainly made by the attending physician, without a pre-established protocol. Third, we did not differentiate between *M*. *bolletii* from *M*. *abscessus*. However, *M*. *bolletii* is a very rare subspecies of MABC in South Korea [33]. Fourth, we did not genotype the isolates, so we were unable to distinguish between relapse and reinfection in patients who experienced reversion. Finally, the phenotypic azithromycin susceptibility against the MABC isolates was not provided in our clinical routine testing.

In conclusion, we found that approximately one-fourth of patients with MABC lung disease who did not receive treatment for at least 2 years after diagnosis experienced spontaneous conversion, suggesting that a watchfully waiting approach, without immediate treatment initiation, could be acceptable for some patients with MABC lung disease with a stable clinical course. However, continuous follow-up is needed for these patients because not a few patients could have reversion after spontaneous sputum conversion.

## Supporting information

S1 Table Clinical characteristics of 18 patients with spontaneous conversion based on reversion. (DOCX)
