# Supplementary material for: Spontaneous sputum conversion and reversion in Mycobacterium abscessus complex lung disease
Source: PLoS One. 2020 Apr 27;15(4):e0232161. doi: 10.1371/journal.pone.0232161 (PMC7185584; doi:10.1371/journal.pone.0232161)
Supplement: S1 Table — (DOCX) [file pone.0232161.s001.docx]

**Spontaneous sputum conversion and reversion in Mycobacterium abscessus complex lung disease**

**S1 Table** Clinical characteristics of 18 patients with spontaneous conversion based on reversion

| Characteristics | Total (n = 18) | Persistent conversion (n = 13) | Reversion (n = 5) | *P* value |
| --- | --- | --- | --- | --- |
|  |  |  |  |  |
| Age (years) | 60.4 ± 12.5 | 60.2 ± 11.4 | 61.0 ± 16.6 | 0.911 |
| Female gender | 11 (61.1%) | 8 (61.5%) | 3 (60.0%) | > 0.99 |
| Body mass index (kg/m^2^) | 21.0 ± 2.6 | 21.6 ± 2.7 | 19.7 ± 1.9 | 0.178 |
| Current or past smoker | 5 (27.8%) | 3 (23.1%) | 2 (40.0%) | 0.583 |
| Diabetes mellitus | 1 (5.6%) | 0 | 1 (20.0%) | 0.278 |
| Previous history of TB treatment | 6 (33.3%) | 4 (30.8%) | 2 (40.0%) | > 0.99 |
| Previous history of NTM treatment | 4 (22.2) | 3 (23.1%) | 1 (20.0%) | > 0.99 |
| Comorbidities |  |  |  |  |
| Chronic obstructive lung disease | 2 (11.1%) | 1 (7.7%) | 1 (20.0%) | 0.490 |
| Chronic liver disease | 2 (11.1%) | 1 (7.7%) | 1 (20.0%) | 0.490 |
| Diabetes mellitus | 1 (5.6%) | 0 | 1 (20.0%) | 0.278 |
| Etiology |  |  |  | 0.326 |
| *Mycobacterium abscessus* | 7 (38.9%) | 4 (30.8%) | 3 (60.0%) |  |
| *Mycobacterium massiliense* | 11 (61.1%) | 9 (69.2%) | 2 (40.0%) |  |
| Type of disease |  |  |  | 0.218 |
| Fibrocavitary | 1 (5.6%) | 1 (7.7%) | 0 |  |
| Cavitary nodular bronchiectatic | 1 (5.6%) | 0 | 1 (20.0%) |  |
| Noncavitary nodular bronchiectatic | 16 (88.9%) | 12 (92.3%) | 4 (80.0%) |  |
| Positive AFB smear at treatment initiation | 7 (38.9%) | 6 (46.2%) | 1 (20.0%) | 0.596 |
| The presence of cavity | 2 (11.1%) | 1 (7.7%) | 1 (20.0%) | 0.490 |
| Number of involved lobes | 2.2 ± 1.3 | 2.2 ± 1.5 | 2.4 ± 0.5 | 0.289 |

Data are reported as mean ± standard deviations or number (%).

TB, tuberculosis; NTM, nontuberculous mycobacterium; AFB, acid-fast bacilli.
